# Cooperative Role of Thrombopoietin and Vascular Endothelial Growth Factor-A in the Progression of Liver Cirrhosis to Hepatocellular Carcinoma

**DOI:** 10.3390/ijms22041818

**Published:** 2021-02-12

**Authors:** Barbara Vizio, Ornella Bosco, Ezio David, Gian Paolo Caviglia, Maria Lorena Abate, Martina Schiavello, Angela Pucci, Antonina Smedile, Gianluca Paraluppi, Renato Romagnoli, Enrico Lupia, Graziella Bellone, Giuseppe Montrucchio

**Affiliations:** 1 Department of Medical Sciences, University of Turin, Via Genova 3, 10126 Turin, Italy; barbara.vizio@unito.it (B.V.); ornella.bosco@unito.it (O.B.); martina.schiavello@unito.it (M.S.); enrico.lupia@unito.it (E.L.); graziella.bellone@unito.it (G.B.); 2 Pathology Unit, AOU Città della Salute e della Scienza di Torino, University of Turin, Via Santena 7, 10126 Turin, Italy; ezio.david@unito.it; 3 Department of Medical Sciences, University of Turin, Via Cavour 31, 10123 Turin, Italy; caviglia.giampi@libero.it (G.P.C.); marialorena.abate@unito.it (M.L.A.); antonina.smedile@unito.it (A.S.); 4 Department of Histopathology, Pisa University Hospital, Via Roma 57, 56126 Pisa, Italy; apucci@ao-pisa.toscana.it; 5 General Surgery 2U and Liver Transplantation Center, AOU Città della Salute e della Scienza di Torino, University of Turin, Corso Bramante 88, 10126 Turin, Italy; gianluca.paraluppi@unito.it (G.P.); renato.romagnoli@unito.it (R.R.)

**Keywords:** angiogenesis, hepatocellular carcinoma, thrombopoietin, tumor microenvironment, vascular endothelial growth factor-A

## Abstract

Primary thrombopoietic mediator thrombopoietin (THPO) is mainly produced by the liver; it may act as a growth factor for hepatic progenitors. Principal angiogenesis inducer vascular endothelial growth factor-A (VEGF-A) is critical for the complex vascular network within the liver architecture. As a cross-regulatory loop between THPO and VEGF-A has been demonstrated in the hematopoietic system, the two growth factors were hypothesized to cooperatively contribute to the progression from liver cirrhosis (LC) to hepatocellular carcinoma (HCC). The mRNA and protein expression levels of THPO, VEGF-A, and their receptors were examined, compared, and correlated in paired cancerous and LC tissues from 26 cirrhosis-related HCC patients, using qRT-PCR and immunohistochemistry. THPO and VEGF-A were alternatively silenced by small interfering RNA (siRNA) in human liver cancer cell lines Huh7 and HepG2. THPO and VEGF-A expressions significantly increased in tumor versus LC tissues. HCC and paired LC cells expressed similar levels of THPO receptor (R), whereas vascular endothelial growth factor receptor (VEGFR) -1 and VEGFR-2 levels were higher in HCC than in corresponding LC tissue samples. A significant linear correlation emerged between THPO and VEGF-A transcripts in HCC and, at the protein level, THPO and THPOR were significantly correlated with VEGF-A in tumor tissues. Both HCC and LC expressed similar levels of gene and protein hypoxia inducible factor (HIF)-1α. Positive cross-regulation occurred with the alternative administration of siRNAs targeting THPO and those targeting VEGF-A in hypoxic liver cancer cell lines. These results suggest THPO and VEGF-A might act as interdependently regulated autocrine and/or paracrine systems for cellular growth in HCC. This might be clinically interesting, since new classes of THPOR agonistic/antagonistic drugs may provide novel therapeutic options to correct the frequent hemostatic abnormality seen in HCC patients.

## 1. Introduction

Hepatocellular carcinoma (HCC) is the most prevalent primary liver malignancy, ranking as the third-highest cause of cancer deaths worldwide. The tumor is characterized by highly heterogenetic pathogenesis, with aggressive clinical course, poor prognosis, and short survival, due to intrahepatic spread and extrahepatic metastasis [1]. Hepatic resection, liver transplantation, and local ablation have improved the outcome in early-stage disease, but tumor recurrence after treatment is high. To date, no effective therapy exists for patients diagnosed with advanced-stage HCC [2].

HCC is a classic example of an inflammation-triggered malignancy, with more than 90% of cases arising as the end stage of persistent inflammation and chronic liver disease, with hepatitis B or C virus infections, alcoholic liver disease, and non-alcoholic fatty liver disease as the main causes underlying the formation and progression of liver cirrhosis (LC) [3].

In order to identify and characterize early tumors, which may have a better prognosis than larger lesions, and to develop more effective therapeutic approaches, it is vital to clarify the molecular events governing tumor initiation and progression in the context of chronic liver injury, such as LC.

The liver is a major site of thrombopoietin (THPO) production, with hepatocytes being the most important cellular source of this growth factor [4]. THPO, initially identified as the primary growth factor responsible for regulating megakaryopoiesis and platelet production [5], possesses wide-ranging biological effects. It enhances the response of mature platelets to activating events [6] and, either in synergy with other hematopoietic cytokines [7] or by activating a vascular endothelial growth factor-A (VEGF-A) internal autocrine loop, supports the survival and expansion of hematopoietic stem cells [8]. Moreover, the THPO/THPO receptor (R) axis plays an important role in the development and progression of chronic myelo-proliferative neoplasms [9]. Most recently, THPO has been shown to play important and nonredundant paracrine/autocrine roles in other cellular systems, including human endothelial cells [10], liver sinusoidal endothelial cells in mice [11], hepatic progenitor cells in rats [12], and tumor cells from human lung, stomach, liver, and thyroid [13], suggesting a possible relationship between cancer and hemostatic disorders. Therefore, in the tumor microenvironment, THPO not only controls the number and activity of platelets, recognized as promoters of tumor growth and dissemination at different levels [14], by directly interacting with its surface receptor c-Mpl (THPOR) [15] expressed by endothelial and tumor cells, it may also activate multiple intracellular pathways, leading to the induction of new vessel formation [10], cell migration, and chemoinvasion [16].

Angiogenesis and the acquisition of self-sustaining growth are the most important early events in the neoplastic process. VEGF-A, which induces intracellular signaling through the cell-surface-bound receptors vascular endothelial growth factor receptor (VEGFR)-1 (Flt-1) and VEGFR-2 (Flk-1/KDR) [17], has been identified as a crucial mediator regulating physiological and pathological angiogenesis [18] and, in some instances, survival and repopulation of tumor cells [19,20].

A common feature of liver diseases is tissue hypoxia due to an imbalance of metabolic demand and supply. The cells adapt to the low oxygen levels through the hypoxia-inducible factor (HIF) signaling pathway. In chronic liver disease, prolonged HIF-mediated adaptive responses to tissue hypoxia may be detrimental through accelerating fibrosis development and promoting tumor cell growth and metastasis [21]. In hematopoietic system, THPO may induce VEGF-A production by promoting HIF-1α stabilization and activation [8]. THPO’s contribution and its possible interplay with VEGF-A in the signaling that occurs in HCC development in the context of LC is still unknown.

This study examines and compares expression patterns of THPO, VEGF-A, their relative receptors, and HIF-1α in cancerous and cirrhotic liver cells from subjects with HCC arising against a background of LC. It could support the hypothesis that, under hypoxic stress, a possible novel mechanism, based on THPO and VEGF-A interaction, might be critical in promoting HCC development from LC, thereby shedding light on predictive markers and targets for biological therapies of HCC.

## 2. Results

### 2.1. THPO and THPOR Transcript Expression in HCC and in Paired LC Tissues

**Figure 1:**
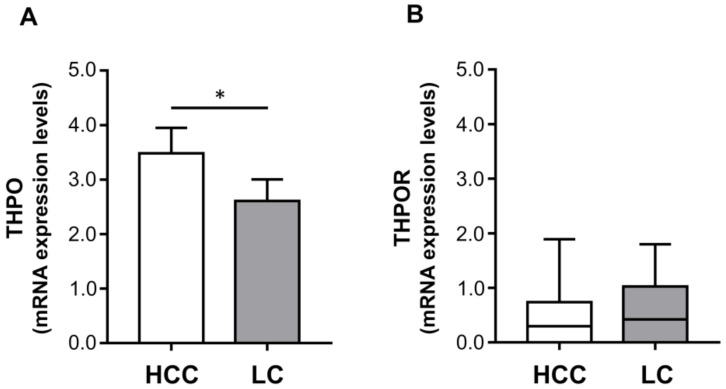
The mRNA expression levels of thrombopoietin (THPO) (**A**), and THPO receptor (R) (**B**). The mRNA levels were assessed in hepatocellular carcinoma (HCC) and paired liver cirrhosis (LC) tissues (*n* = 26) via qRT-PCR and normalized to housekeeping genes. Mean ± SEM (**A**) or median (range) (**B**) gene expression values are shown as a column bar or box plot, respectively. The *p* values were obtained by paired *t* test (* *p* < 0.05) (**A**) or Wilcoxon signed rank test (not statistically significant) (**B**).

Hepatocytes are the principal THPO producers, and some HCC cell lines express THPOR [22]. Thus, to clarify the possible role of THPO/THPOR signaling in the development of HCC, the transcripts for THPO and THPOR (c-Mpl) were examined in 26 HCC tissues and in their cirrhotic counterparts. THPO mRNA levels were significantly higher in tumor specimens than in LC tissues from the same patient (mean ± SEM: 3.48 ± 0.46 vs. 2.61 ± 0.40 (Figure 1A).

Conversely, similar THPOR transcript expression was found in tumor and LC areas in all cases (median (range) expression levels: 0.30 (0–1.89) vs. 0.42 (0–1.80)) with no significant difference between the two (Figure 1B).

### 2.2. THPO and THPOR Protein Expression in HCC and in Paired LC Tissues

**Figure 2:**
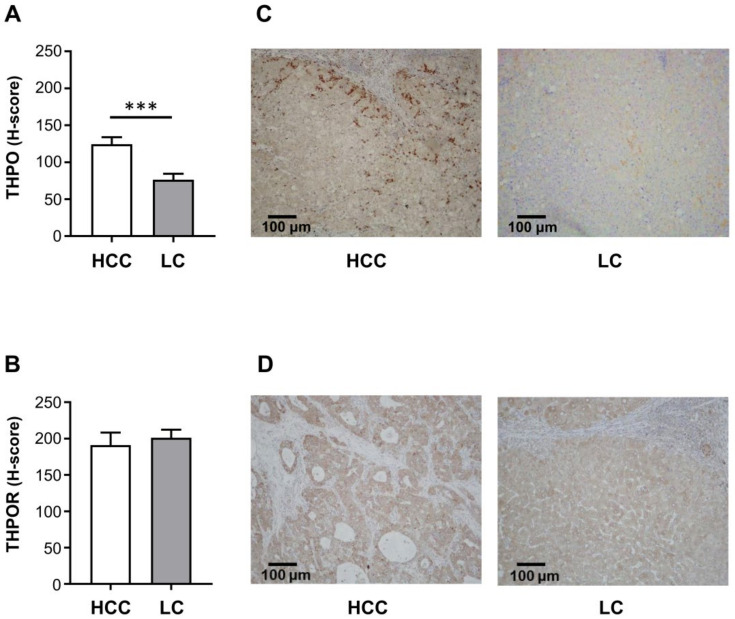
Protein expression of THPO (**A**) and THPOR (**B**). IHC was run in tumoral and paired cirrhotic liver tissue specimens (*n* = 26). Semi-quantitative evaluation was by H-score, expressed as mean ± SEM. Specimens (**C**,**D**) are representative examples of the 26 HCC and paired LC tissue samples (original magnification 100×). The *p* values were obtained by paired *t* test (*** *p* < 0.001 (**A**) and not statistically significant (**B**)).

The hepatocyte expression of THPO protein was evaluated by IHC in HCC and corresponding LC samples; THPO protein levels were much higher in tumor cells than in associated cirrhotic liver, (mean ± SEM H-score: 124.50 ± 9.31 vs. 76.40 ± 8.05) (Figure 2A). Immunoreactivity was located mainly at the tumor-infiltrating border, rather than in the central core of neoplastic proliferation, i.e., at the contact point between neoplastic epithelial cells and fibrotic stroma (Figure 2C). Consistently with the mRNA analysis, THPOR immunoreactivity was similar in HCC and paired LC hepatocytes (mean ± SEM H-score: 191.00 ± 17.21 vs. 201.10 ± 11.06) (Figure 2B). Representative images of IHC staining in HCC and LC tissue samples are shown in Figure 2C,D.

### 2.3. VEGF-A, VEGFR-1, and VEGFR-2 Transcript Expression in HCC and Paired LC Tissues

**Figure 3:**
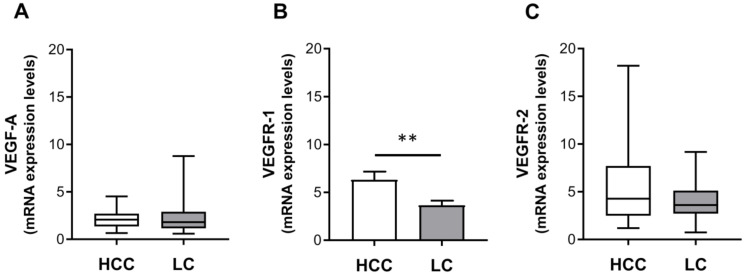
The mRNA expression levels of vascular endothelial growth factor-A (VEGF-A) (**A**), vascular endothelial growth factor receptor (VEGFR)-1 (**B**), and VEGFR-2 (**C**). The mRNA levels were assessed in HCC and paired LC tissues (*n* = 26) via qRT-PCR and normalized to housekeeping genes. Median (range) (**A**,**C**) or mean ± SEM (**B**) gene expression values are shown as a box plot or column bar, respectively. The *p* values were obtained by Wilcoxon signed rank test (**A**,**C**) (not statistically significant) or paired *t* test (**B**) (** *p* < 0.01).

THPO has been shown to drive VEGF-A expression in THPOR/c-Mpl-expressing hematopoietic immature cells or cell lines [23], thus local expression levels of VEGF-A were examined in paired HCC and cirrhotic tissues. VEGF-A mRNA expression was quantitatively detected in all 26 (100%) paired HCC and LC tissue samples. In HCC tissues, expression levels ranged from 0.66 to 4.53, median 2.08, while in LC tissues they ranged from 0.59 to 8.78, median 1.81. However, no significant difference in VEGF-A expression between HCC and paired LC tissues was found (Figure 3A).

The possible activation of the VEGF-A autocrine signaling pathway was evaluated in HCC and matched LC samples, analyzing expressions of VEGFR-1 and VEGFR-2 mRNA levels. All paired tissue samples expressed both VEGF receptors. VEGFR-1 transcript was significantly higher in HCC than in the paired LC samples (mean ± SEM expression level: 6.32 ± 0.84 vs. 3.65 ± 0.50) (Figure 3B). VEGFR-2 transcript tended to be higher in HCC than in paired LC samples (median (range) expression level: 4.28 (1.19–18.20) vs. 3.60 (0.74–9.18)), but at the limit of statistical significance (*p* = 0.069) (Figure 3C).

### 2.4. VEGF-A, VEGFR-1 and VEGFR-2 Protein Expression in HCC and in Paired LC Tissues

**Figure 4:**
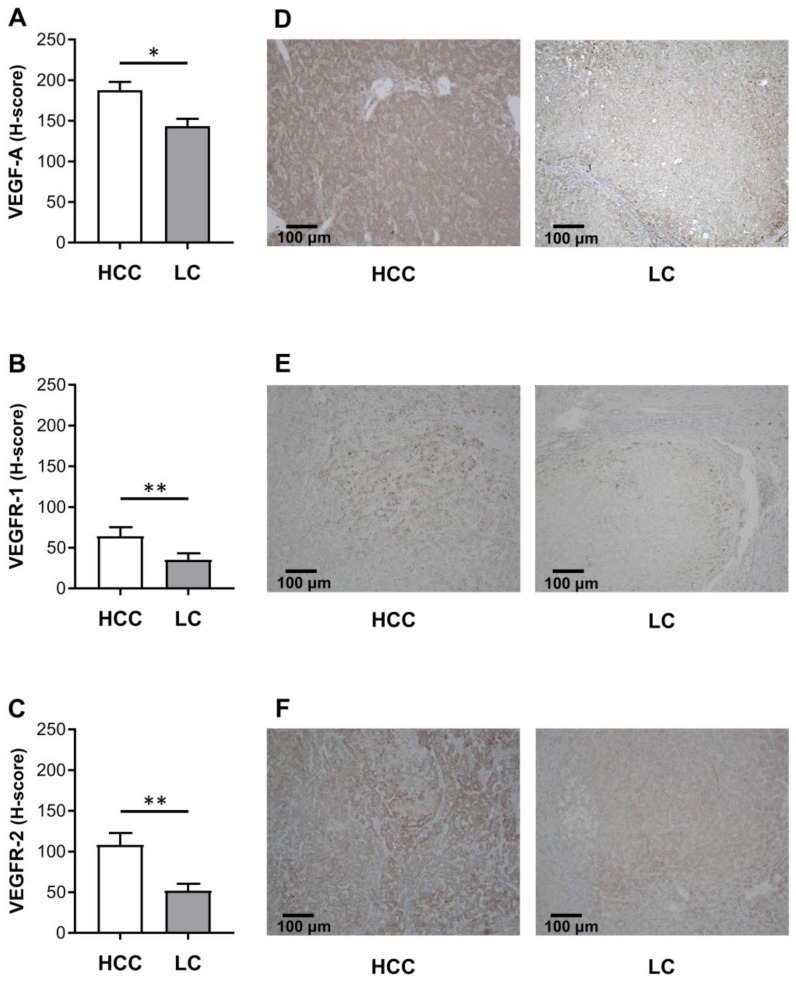
Protein expression of VEGF-A (**A**), VEGFR-1 (**B**), and VEGFR-2 (**C**). IHC was run in tumoral and cirrhotic liver tissue specimens (*n* = 26). Semi-quantitative evaluation by H-score, expressed as mean ± SEM. Specimens (**D**–**F**) are representative examples of 26 HCC and 26 paired LC tissue samples (original magnification 100×). Statistical analysis included data on all patients. The *p* values were obtained by paired *t* test (* *p* < 0.05, ** *p* < 0.01).

The parallel immunohistochemical study of VEGF-A protein expression showed that, in both cancer cells and cirrhotic hepatocytes, staining was mainly located in the cytoplasm, and was significantly higher in HCC than in paired LC tissues (mean ± SEM H-score: 187.70 ± 10.20 vs. 143.40 ± 9.06) (Figure 4A). All tumors and cirrhotic counterparts were positive for VEGF-A.

VEGFR-1 protein expression occurred in 23 of 26 HCC samples (89%) and in 24 of 26 LC samples (92%). Three HCC specimens were negative for VEGFR-1 (11%); the receptor was expressed in the corresponding LC areas. Two LC specimens were negative for VEGFR-1 (8%); the receptor was expressed in the matched malignant tissue samples. Stronger VEGFR-1 granular staining of the cytoplasm was observed in positive HCC cells than in LC lesions (mean ± SEM H-score: 64.26 ± 10.91 vs. 35.23 ± 7.94) (Figure 4B). Conversely, VEGFR-2 protein expression occurred in all 26 (100%) HCC and in 24 (92%) paired LC tissue samples. VEGFR-2 expression was higher in HCC cells than in nontumorous LC areas (mean ± SEM H-score: 108.10 ± 14.61 vs. 51.87 ± 8.60) (Figure 4C). Immunohistochemically-positive staining was chiefly localized in the cytoplasm of both malignant and cirrhotic hepatic cells. Representative images of IHC staining for VEGF-A, VEGFR-1, and VEGFR-2 in HCC and LC tissue samples are in Figure 4D–F, respectively.

### 2.5. Interrelations between Expression of THPO, VEGF-A, and THPOR in HCC and LC

**Figure 5:**
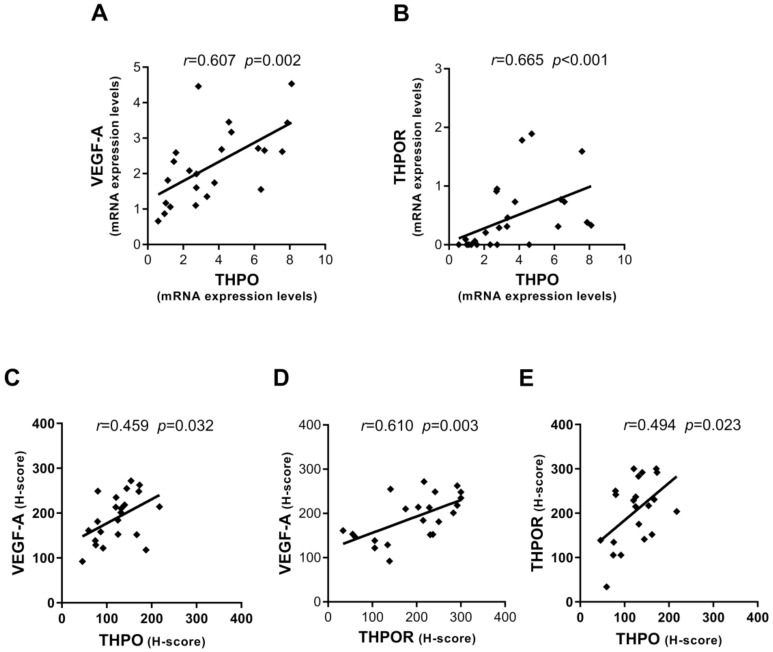
Interrelationships between mRNA and protein expression levels of THPO, VEGF-A, and THPOR in HCC. Correlations between mRNA expression levels of THPO and VEGF-A (**A**) and THPO and THPOR (**B**). Correlations between protein expression in IHC staining (H-score) of THPO and VEGF-A (**C**), THPOR and VEGF-A (**D**), and THPO and THPOR (**E**). Analyses by Pearson (**A**–**E**) or Spearman (**B**) correlation tests; correlation coefficient *r* and *p* values are also shown.

Possible interrelationships between THPO/THPOR and VEGF-A/VEGFRs in the context of oncogenic transformation occurring in HCC have never been explored. Thus, this aspect was investigated by analyzing the correlation between gene expression and protein level in paired tumor and LC tissues; only statistically significant correlations are presented here. A consistent positive correlation was observed in HCC between THPO and VEGF-A transcripts (Figure 5A), as well as between THPO and THPOR mRNA (Figure 5B).

With regard to protein expression evaluated by IHC staining of hepatocytes in HCC tissue, both THPO and THPOR were found to be significantly correlated with VEGF-A (Figure 5C,D). Moreover, a positive statistically significant correlation was observed between THPO and THPOR (Figure 5E).

**Figure 6:**
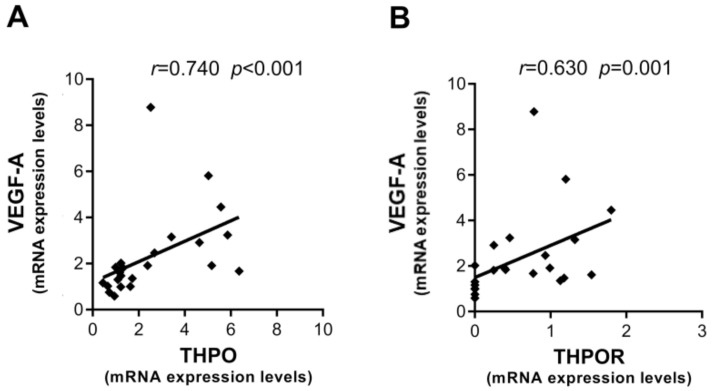
Interrelationships between mRNA expression levels of THPO, VEGF-A, and THPOR in LC. Correlations between mRNA expression levels of THPO and VEGF-A (**A**) and THPOR and VEGF-A (**B**). Analyses by Spearman correlation test; correlation coefficient *r* and *p* values are also shown.

By contrast, in LC positive correlations were only found at the mRNA level between VEGF-A and the THPO/THPOR axis (Figure 6A,B).

### 2.6. HIF-1α mRNA and Protein Expression in HCC and in Paired LC Tissues

**Figure 7:**
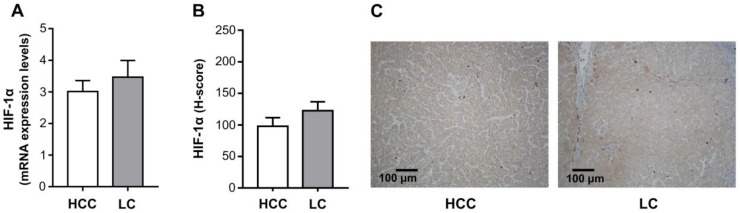
Hypoxia inducible factor (HIF)-1α mRNA (**A**) and protein (**B**) expression levels. The mRNA levels were assessed in HCC and paired LC tissues (*n* = 26) via qRT-PCR and normalized to housekeeping genes. IHC was run in tumoral and cirrhotic liver tissue specimens (*n* = 26). Mean ± SEM gene expression values and H-score are shown as column bars. The statistical analysis was conducted using a paired *t* test (not statistically significant). Representative examples of IHC staining for HIF-1α in HCC and paired LC tissue samples (original magnification 100×) (**C**).

It has been shown that, in the hemopoietic system, THPO enhances autocrine expression of VEGF-A through a hypoxia inducible factor-1α (HIF-1α)-dependent pathway [8]. To verify whether this mechanism is also involved during the transition from LC to HCC, HIF-1α expression in paired tumor and cirrhotic tissue samples was analyzed at both the mRNA and protein levels. As shown in Figure 7, the HIF-1α gene was similarly expressed in HCC and paired LC tissues (mean ± SEM expression level: 3.01 ± 0.35 vs. 3.47 ± 0.53) (Figure 7A), as was the protein in malignant and cirrhotic hepatocytes (mean ± SEM H-score: 97.91 ± 13.63 vs. 122.50 ± 14.02) (Figure 7B). Representative images of IHC staining in HCC and LC tissue samples are shown in Figure 7C. However, in HCC, HIF-1α only correlated with VEGFR-2 at the mRNA expression level, and only with VEGF-A at the protein level (*r* = 0.563 *p* = 0.023, *r* = 0.509 *p* = 0.013, respectively; Pearson correlation test).

### 2.7. THPO and VEGF-A Cross-Regulation in HCC Cell Lines Grown in Hypoxic Conditions

**Figure 8:**
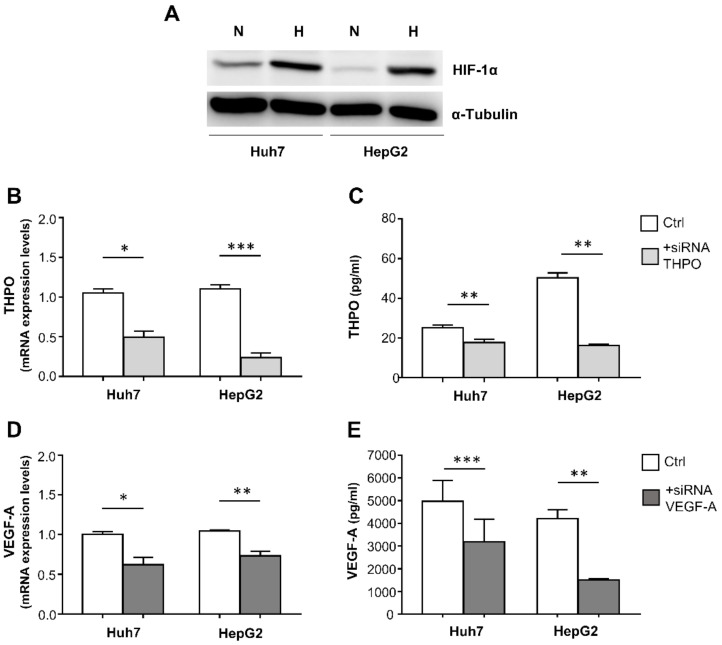
Hypoxia (H) induces an increase in HIF-1α protein, detected by Western blotting, compared with normoxia (N) in Huh7 and HepG2 cells (**A**). THPO and VEGF-A silencing in hypoxic Huh7 and HepG2 liver cancer cells: mRNA expression and protein levels were analyzed 72 h after transfection with THPO (**B**,**C**) and VEGF-A (**D**,**E**) siRNA. Results are expressed as mean ± SEM of 4 separate experiments; *p* values were obtained by paired *t* test (* *p* < 0.05, ** *p* < 0.01, *** *p* < 0.001).

To consolidate evidence of possible cross-regulation between THPO and VEGF-A in HCC, siRNA technology was used to analyze the mutual effect on mRNA and protein expression of the alternative ablation of THPO or VEGF-A, using as model two c-mpl expressing liver cancer cell lines, Huh7 and HepG2, grown in a hypoxia chamber (1% O_2_) to mimic the in vivo hypoxic condition of the tumor. Achievement of hypoxia in the cells was demonstrated by a significant increase in HIF-1α protein in comparison with cells grown in standard conditions, as assessed by Western blotting (Figure 8A). The efficiency of the THPO and VEGF-A silencing was determined by analyzing the levels of corresponding mRNA and proteins.

In both cell lines, THPO siRNA effectively reduced THPO mRNA (Figure 8B) and protein (Figure 8C); similarly, VEGF-A siRNA resulted in the down-regulation of both VEGF-A mRNA (Figure 8D) and protein (Figure 8E).

**Figure 9:**
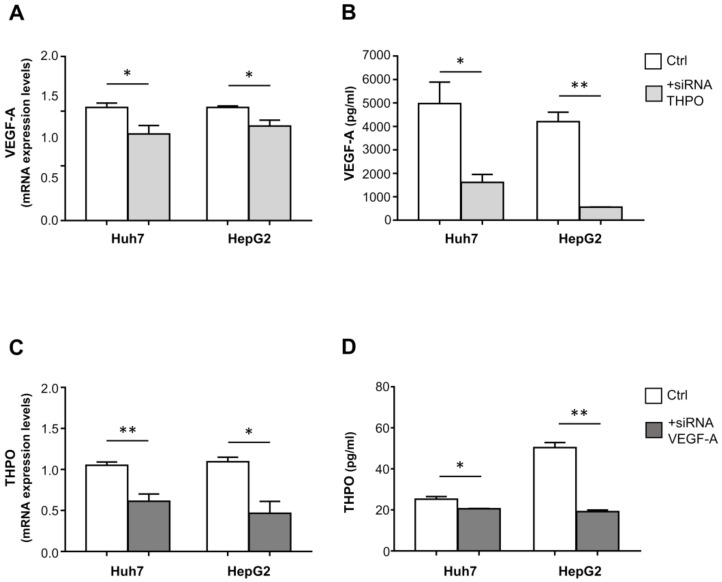
THPO and VEGF-A silencing cross-effects on VEGF-A and THPO transcripts and proteins in hypoxic Huh7 and HepG2 liver cancer cells. mRNA expression and protein levels of VEGF-A and THPO analyzed after transfection with THPO (**A**,**B**) and VEGF-A (**C**,**D**) siRNA, respectively. Results are expressed as mean ± SEM of 4 separate experiments. Gene expression values or protein levels are shown as column bars. The *p* values were obtained by paired *t* test (* *p* < 0.05, ** *p* < 0.01).

Importantly, in both the Huh7 and HepG2 cell lines, transfection with THPO-siRNA knocked down the endogenous level of VEGF-A transcript (Figure 9A) and protein (Figure 9B); conversely, VEGF-A siRNA-transfected cells exhibited a marked reduction in both THPO mRNA (Figure 9C) and protein (Figure 9D).

## 3. Discussion

Recent studies suggest that growth factors produced by tumor cells can generate local insidious loops that may produce self-perpetuating signals and/or a tumor-favoring microenvironment [24].

The present innovative study targeted expression of THPO, VEGF-A, and their receptors on hepatocytes in HCC and matched cirrhotic tissues, and showed that: (i) hepatic THPO production increased in the transition from cirrhosis to liver cancer; (ii) in the tumor context, but not in LC, the THPO, THPOR, and VEGF-A protein expressions were positively correlated; (iii) HCC and LC expressed comparable levels of HIF-1α at both mRNA and protein levels; and (iv) siRNA-mediated knock-down of THPO and VEGF-A signaling in hypoxic hepatic cancer cell lines Huh7 and HepG2, and induced significant cross-inhibitory effects on both mRNA and protein expression. These results suggest that interdependence between THPO and VEGF-A exists in HCC, creating a favorable shift toward tumor growth and expansion in an angiogenesis dependent/independent manner.

This comparative study found that THPO expression was significantly higher in HCC tissues than in their cirrhotic counterparts, at both the mRNA and protein levels, with THPO-producing malignant hepatocytes mainly localized at stroma contact sites, where THPO target cells, including vascular cell, platelet, and cancer-stem-cell niches, might potentially create a microenvironment conductive to tumorigenesis, angiogenesis, and metastatic spread.

By contrast, HCC and paired LC cells expressed comparable levels of THPOR, but only in HCC, a significant positive correlation was found between THPO and THPOR at both mRNA and protein levels. In rats, hepatic progenitors of fetal and adult liver, but not hepatocytes, express THPOR on the cell surface [12]. In humans, adult equivalents of these hepatic progenitor cells have been identified as a quiescent cell subset, acting as a reserve population during repair and regeneration processes in the injured liver. Several lines of evidence indicate that THPO can target regenerating and malignant hepatocytes. The presence of hepatocyte-like cells of intermediate differentiation in cirrhotic nodules, sites of ongoing regeneration, has been reported in direct correlation with the degree of inflammation and liver injury [25]. Moreover, established liver cancer cell lines Huh7, HepG2, and Hep3B express functionally active THPOR, and interaction with recombinant THPO generates cell migration and chemoinvasion [16,26].

Based on these and others’ results, it may be assumed that, under particular conditions such as cancer and LC, liver tissue may be a site of responsive THPOR expression. In the setting of chronic, unresolved liver injury, such as LC, the molecular mechanisms driving liver repair may similarly trigger the initiation and promotion of hepatocarcinogenesis, by binding THPO to the cognate receptor expressed by regenerating hepatocytes in dysplastic nodules. In the HCC context, the increased THPO level appears to be a novel mediator capable of modifying the biology of malignant THPOR-positive hepatocytes, by triggering signaling pathways with tumor progression activities. However, the effective contribution of the THPO/THPOR axis in the progression from cirrhosis to HCC remains to be clarified.

VEGF-A is considered to be the principal angiogenesis-stimulating factor, and to be correlated with tumor neovascularization, tumor invasion, and metastasis, including in HCC [14,15,16]. In accordance with a previous report [27], similar expression values of VEGF-A mRNA were found in HCC and in paired cirrhotic liver lesions. However, when the analysis was restricted to hepatocytes employing IHC to determine protein content, VEGF-A-positive expression was higher in HCC, suggesting the increase of VEGF-A production by hepatocytes, from adjacent cirrhotic liver to tumor, may be stepwise. Hepatic vascular proliferation with pathological angiogenesis has been reported to be enhanced in HCC in comparison with chronic hepatitis and LC [28].

Regarding VEGF-A receptors, the present study found higher expression of VEGFR-1 mRNA in HCC tissue and protein in malignant hepatocytes than in corresponding cirrhotic liver. The direct VEGFR-1-mediated pro-angiogenic activity is usually weak, but the receptor appears to trigger a pathway in cancer cells that induces epithelial–mesenchymal transition, the critical process for acquiring invasive potential and promoting cancer-cell metastasis in vivo [29]. A number of studies have reported expression of VEGFR-1 and its prognostic significance in multiple human malignancies, including HCC [30,31]. The present results show the presence of the VEGF-A/VEGFR-1 axis to be more marked in malignant liver, suggesting the tumor might play an important role in acquiring a growth advantage via an autocrine mechanism.

The present study also found that, at the protein level, VEGFR-2 was significantly higher in HCC than in cirrhotic liver hepatocytes. Although VEGFR-2 has a predominantly angiogenic role, its presence on hepatocytes implies autocrine VEGF-A signaling, favoring the progression of HCC carcinogenesis. An experimental model found that VEGF-A enhanced HCC proliferation by interacting with VEGFR-1 and VEGFR-2, and that treatment of high VEGFR-1/2-expressing HepG2 cells with sorafenib, an inhibitor targeting several kinases including VEGFRs, inhibited cell proliferation, reduced VEGFR-2 mRNA expression in vitro, and delayed xenograft tumor growth in vivo [32].

The interconnections between THPO and VEGF-A signaling in the oncogenic transformation occurring in HCC are still unknown. The present study found a significant linear correlation between the VEGF-A protein and THPO/THPOR axis, in HCC tissues but not in LC.

It has been reported that THPO, by enhancing the stability of HIF-1α protein [8,33], which is the primary transcriptional mediator of the hypoxic response [34], may cause a marked rise in VEGF-A release in hematopoietic progenitor cells and in cell lines that express THPOR/c-Mpl [23]. Conversely, in the bone marrow stromal cell line MS-5, VEGF-A can increase expression of THPO [35].

**Figure 10:**
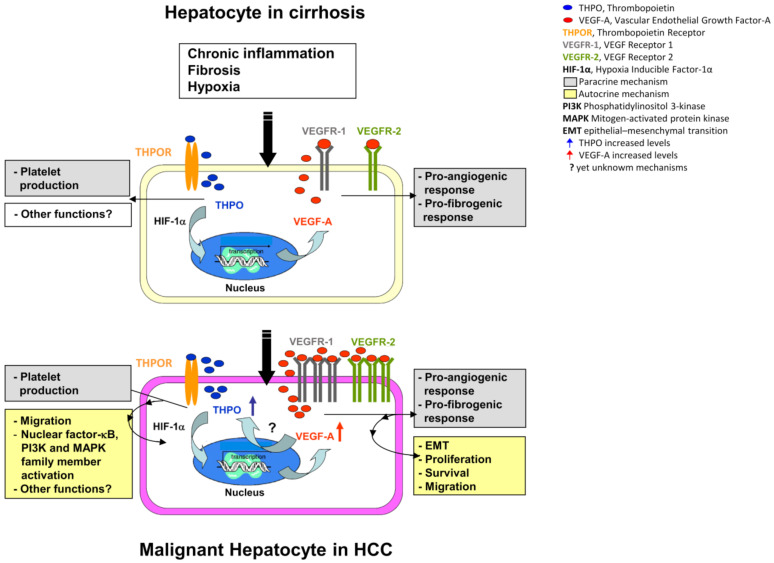
Hypothetical role of THPO in the transition from liver cirrhosis to HCC. In HCC, THPO binds hepatocytes c-Mpl, activates HIF-1α, and stimulates the production of VEGF-A. VEGF-A acts in an internal autocrine fashion, and by a still-unknown mechanism, induces the production of THPO, creating an insidious positive mutual loop that provides signals for progression from preneoplastic liver lesions to tumor.

The finding that both HCC and LC express similar levels of both gene and protein HIF-1α is not surprising. Fibrinogenesis, resulting from liver injury and cirrhosis, leads to a reduction in vascularization, which contributes to tissue hypoxia. In HCC, typically arising in the setting of cirrhosis, the same phenomenon occurs due to the rapid growth of the tumor [36]. However, correlation analysis shows that HIF-1α is positively correlated with tissue VEGFR-2 at the mRNA level only in HCC, and with VEGF-A proteins in malignant hepatocytes. It may thus be speculated that, in a comparable hypoxic situation, the preneoplastic setting of the cirrhotic background provides a conducive environment predisposing transformed hepatocytes to become high producers of THPO. THPO, in turn, by stabilizing HIF-1α, induces VEGF-A dependent autocrine/paracrine loops, acting on VEGFR-2 expressing tumor, or on non-tumor cells that may affect HCC development and progression (Figure 10).

Conversely, enhanced activity of the VEGF-A/VEGFR-2 ligand/receptor complex in the context of HCC, by other still-unknown oxygen-independent mechanisms, may increase THPO expression by malignant hepatocytes, triggering a positive autocrine feedback loop that augments pro-tumorigenic signal propagation. This assumption is demonstrated by the mRNA and protein cross-inhibition between THPO and VEGF-A, which has been observed in hypoxic Huh7 and HepG2 cells to mimic in vivo tumor condition, upon cell transfection with VEGF-A-siRNA and THPO-siRNA.

Taken together, these findings may be of clinical interest. Several therapeutic options for treating early/intermediate and late stage HCC are based on inhibiting angiogenesis, in particular by multi-kinase inhibitors and antibodies, and by peptibody-targeting angiogenesis factors [37]. Conventional treatment options employing antiangiogenic approaches entail starving tumors of their blood supply. However, in light of the present findings, recreating hypoxic conditions could retrigger the deleterious THPO/VEGF-A positive feedback, favoring tumor growth. Conversely, it has been shown that blockading the VEGF receptor with the specific inhibitor SU5416 substantially blunts THPO-induced growth in Mpl positive-primitive hematopoietic cells [8]. It is not yet clear to what extent action mechanisms, dosing, timing, and administration schedules of the different antiangiogenic compounds actually impact clinical endpoints.

Traditional treatment options to correct thrombocytopenia, found in most patients with chronic liver disease (CLD) and in some with HCC, are invasive and highly risky [38]. Initial studies on the recently developed recombinant THPOR agonists (Eltrombopag, first-generation Romiplostim, and first- and second-generation Avatrombopag) have shown these agents to be beneficial in CLD and HCC patients, especially in improving platelet counts. However, these studies relate to short-term treatment, and the effects have been investigated at the systemic level rather than locally [39]. The results of the present study indicate that, in the transition from cirrhotic to malignant hepatocytes, these cells improve their ability to synthesize THPO by creating paracrine/autocrine loops that may affect tumor-related pathways.

In chronic myelo-proliferative neoplasms, sustained by enhanced THPO/c-Mpl signaling, a THPOR antagonist (LCP4) has been shown to deplete malignant myelofibrosis in hematopoietic stem and progenitor cells [40]. Considering the role played by THPO in favoring HCC development, it is reasonable to assume that the above therapeutic approach could also selectively interfere with pro-inflammatory and tumor-promoting THPO action. For all these reasons, in the authors’ view, curative strategies aimed at manipulating THPOR in HCC patients should be evaluated with extreme care.

In conclusion, although the significance of this preliminary study is somewhat limited by the small patient cohort, it is nevertheless indicative. The VEGF-A/THPO interplay in hepatocarcinogenesis certainly deserves further investigation, not only to clarify its biological role and mode of action, but also to explore its possible clinical impact.

## 4. Materials and Methods

### 4.1. Patients and Tissue Samples

Liver tissue from 26 patients with HCC associated with LC, who underwent partial hepatectomy or liver transplantation between 2011 and 2013 at the Department of General Surgery 2U-Liver Transplantation Center, at A.O.U. Città della Salute e della Scienza di Torino, Italy, were retrospectively evaluated. No patient had received preoperative cancer treatment before surgical resection, and none had apparent distant metastatic disease. Table 1 gives the patients’ clinicopathological details.

HCC and matched distant tumor-free LC tissues, excluding necrotic areas, were collected immediately after surgical resection, stored in RNAlater Solution (Ambion, Thermo Fisher Scientific, Waltham, MA, USA) at −80 °C for RNA isolation, or fixed in 10% formalin and included in paraffin for routine stains (hematoxylin and eosin, Periodic acid–Schiff–diastase, Sirius red, Perl’s) and immunohistochemical analysis. Tissue samples were provided following the local tissue-banking protocol for discarded tissue (#80911) approved by the University of Turin Ethics Board (Comitato Bioetica di Ateneo). Written informed consent was obtained from all patients; all samples were anonymously coded in accordance with the 1975 Declaration of Helsinki.

### 4.2. Cell Line and Cell Culture

The human liver cancer cell lines Huh7, kindly provided by Professor Maurizio Parola (Department of Clinical and Biological Sciences, University of Turin) and HepG2, obtained from American Type Culture Collection (Manassas, VA, USA), were grown in DMEM (Gibco, Thermo Fisher Scientific, Waltham, MA, USA) supplemented with 10% fetal bovine serum (Gibco), 100 IU/mL penicillin, and 100 μg/mL streptomycin at 37 °C in a humidified atmosphere with 5% CO_2_. For the hypoxia experiments, cell lines were cultured in a hypoxia chamber (1% O_2_) for 24 h.

### 4.3. THPO and VEGF-A Silencing and Interaction under Hipoxic Conditions

Huh7 and HepG2 cells were transfected with specific stealth RNAi small interfering RNA (siRNA) (THPO: HSS110737; VEGF-A: HSS111274-Life Technologies, Thermo Fisher Scientific, Carlsbad, CA, USA) or with non-specific siRNA as negative control, at concentrations of 10 nM. Transfection was performed using Lipofectamine RNAiMAX (Life Technologies) following the manufacturer’s instructions; 72 h after transfection, cells were detached, reseeded and maintained under hypoxic conditions (1% O_2_) for 24 h. Cultured supernatants were collected to evaluate THPO and VEGF-A protein expression by ELISA, and total RNA was extracted from cells to determine THPO and VEGF-A gene expression by qRT-PCR.

### 4.4. Quantitative Reverse Transcription-Polymerase Chain Reaction (qRT-PCR)

Total RNA was extracted using the RiboPure™ RNA Purification Kit (Ambion) following the manufacturer’s instructions. The RNA was quantified using a NanoDrop ND 1000 analyzer (NanoDrop Technologies, Wilmington, DE, USA). RNA quality was assessed by microcapillary electrophoresis with an Agilent 2100 Bioanalyzer (Agilent Technologies, Santa Clara, CA, USA). Samples with an RNA integrity number (RIN) ≥6.5 were considered suitable for subsequent analyses. To remove traces of genomic DNA, total RNA was treated with DNase I (Ambion) and subsequently reverse-transcribed using the High-Capacity cDNA Reverse-Transcription Kit with RNase Inhibitor (Applied Biosystems, Thermo Fisher Scientific).

The qRT-PCR was run using probes from Applied Biosystems specific for THPO (Hs01061346_m1), THPOR (Hs00180489_m1), VEGF-A (Hs00900054_m1), VEGFR-1 (Hs01052961_m1), VEGFR-2 (Hs00911700_m1), and HIF-1α (Hs00153153_m1); and for the reference genes EIF4A2 (Hs00756996_g1) and UBC (Hs00824723_m1) for tissue samples; and ACTB (Hs99999903_m1) for cell lines.

The qRT-PCR was performed with the iCycler (BioRad, Hercules, CA, USA) using the TaqMan™ Universal PCR Master Mix, no AmpErase™ UNG (Applied Biosystems) following the manufacturer’s protocol. Data were analyzed with the Gene Expression Analysis for iCycler iQ Real Time PCR Detection System (BioRad). The mRNA expression level was calculated as described by Vandesompele et al. [41], using the equation:

Expression level = relative quantity/normalization factor(1)

In Equation (1), the normalization factor is the geometric mean of the relative quantities for all reference genes having the same identifiers as the sample.

### 4.5. Immunohistochemistry (IHC)

Formalin-fixed, paraffin-embedded serial sections were dewaxed, rehydrated, and subjected to heat-induced epitope retrieval using Target Retrieval Solutions, Citrate pH 6 or Tris/EDTA pH 9 (Dako, Agilent Technologies, Santa Clara, CA, USA), in an electric pressure-cooker for 20 min at 120 °C. To quench endogenous peroxidase activity, slides were incubated in 3% (*v*/*v*) hydrogen peroxide in methanol for 25 min. Nonspecific binding was blocked using 10% (*v*/*v*) fetal bovine serum in phosphate-buffered saline containing 0.1% Triton X100 for 1 h. Sections were incubated at 4 °C overnight with anti-human THPO (Antibodies-Online GmbH, Aachen, Germany), VEGF-A (Santa Cruz Biotech., Dallas, TX, USA), or Flt-1 (Santa Cruz Biotech.) rabbit polyclonal antibodies, or anti-human THPOR (R&D Systems, Minneapolis, MN, USA) or Flk-1 (Santa Cruz Biotech.) mouse monoclonal antibodies, or anti-human HIF-1α (Abcam, Cambridge, UK) rabbit monoclonal antibody. Negative controls were performed with nonimmune serum instead of the primary antibody. The slides were then stained with ImmPRESS Reagent (Vector Laboratories, Burlingame, CA, USA) for 30 min, revealed using the ImmPACT DAB Peroxidase Substrate Kit (Vector Laboratories), and counterstained with Mayer’s hematoxylin.

### 4.6. Evaluation of Staining

Histopathological examination was done by a senior pathologist (ED), who was unaware of the clinical findings. There was no positive staining in negative controls. The intensities of THPO, THPOR, VEGF-A, VEGFR-1, VEGFR-2, and HIF-1α expression were evaluated, using a Nikon ECLIPSE 80i optical microscope (Nikon Corporation, Tokyo, Japan), in hepatocytes alone by a semiquantitative scoring system (H-score) incorporating both the intensity and the distribution of specific staining [30]. Briefly, the staining intensity was quantified by determining the percentage of target cells that were positive at each of the intensity scores: negative = 0, weak = 1, moderate = 2, strong = 3. The scores of THPO, THPOR, VEGF-A, VEGFR-1, VEGFR-2, and HIF-1α expression in each field were calculated by summing the percentages (P) of cells staining at each intensity, multiplied by the weighted staining intensity determined from the following formula:

H-score = ∑Pi(i + 1)(2)

where i = 0, 1, 2, 3 and Pi varies from 0 to 100%.

### 4.7. ELISA Assay

THPO and VEGF-A levels in cultured supernatants were quantified using specific ELISA kits (R&D Systems) following the manufacturer’s instructions. The lower detection limits of the assays were 7.45 pg/mL for THPO and 5 pg/mL for VEGF-A.

### 4.8. Western Blot Analysis

Huh7 and HepG2, cultured in normoxic and hypoxic conditions for 24 h, were lysed for 1 h in ice-cold lysis buffer (RIPA Buffer, Sigma-Aldrich, Merk, Darmstadt, Germany) supplemented with a protease and phosphatase inhibitor cocktail (Thermo Fisher Scientific). Protein extracts (40 µg) were resolved by 6% SDS/PAGE under reducing conditions, and electroblotted onto nitrocellulose membranes blocked in TRIS-buffered saline containing 0.3% Tween 20 and 5% bovine serum albumin. Filters were then reacted overnight with anti-human HIF-1α rabbit monoclonal antibody (1:500) and incubated with horseradish peroxidase (HRP)-labeled goat anti-rabbit IgG secondary antibody (1:10,000, Invitrogen, Thermo Fisher Scientific). The immunocomplexes were visualized using an ECL-immunoblotting detection kit (PerkinElmer, Waltham, MA, USA). To confirm equal protein loading, blots were stripped and re-blotted with an anti-α-Tubulin mouse monoclonal antibody (1:2000; Sigma-Aldrich), and then probed with a goat anti-mouse IgG HRP-linked antibody (1:25,000; Bethyl Laboratories Inc., Montgomery, TX, USA).

### 4.9. Statistical Analysis

Statistically significant differences between datasets were evaluated using the Wilcoxon and paired *t*-tests. Relationships between variables were investigated using the Spearman or Pearson correlation tests; *p* values < 0.05 were considered statistically significant. The statistical analysis was performed with the GraphPad Prism 7 package (GraphPad Software, La Jolla, CA, USA).

## Figures and Tables

**Table 1:** Patients’ demographic and histopathological characteristics.

Characteristics	All Patients (*n* = 26)
Gender, male/female, *n*	22/4
Age (years), mean ± SD	61.8 ± 7.8
Platelet count (10^9^/L), mean ± SD	118 ± 76
Albumin (g/dL), mean ± SD	3.45 ± 0.61
Bilirubin (mg/dL), mean ± SD	2.24 ± 2.81
Creatinine (mg/dL), mean ± SD	0.91 ± 0.21
INR, mean ± SD	1.24 ± 1.27
AST (UI/mL), mean ± SD	73 ± 58
ALT (UI/mL), mean ± SD	52 ± 36
**Child–Turcotte–Pugh score**	***n***
A	14
B	9
C	3
MELD, mean ± SD	11 ± 5
**Etiology**	***n***
Viral hepatitis (HBV, HCV)	15
Alcohol-toxic	9
Cryptogenic	2
**Surgical data**	***n***
Partial hepatectomy	11
Liver transplantation	15
**Tumor size and vascular invasion (T)**	***n***
T1	6
T2	15
T3	5
**Grading (G)**	***n***
G1	1
G1/G2	2
G2	17
G2/G3	1
G3	4
G4	1
**BCLC stage**	***n***
A	9
B	4
C	9
D	4

ALT, alanine aminotransferase; AST, aspartate aminotransferase; BCLC, Barcelona Clinic Liver Cancer; HBV, hepatitis B virus; HCV, hepatitis C virus; INR, international normalized ratio; MELD, Model for End-stage Liver Disease; SD, standard deviation.

## Data Availability

The data presented in this study are available on request from the corresponding author. The data are not publicly available due to privacy.

## Abbreviations

CLD	Chronic liver diseases
HCC	Hepatocellular carcinoma
HIF	Hypoxia-inducible factor
HRP	Horseradish peroxidase
IHC	Immunohistochemistry
LC	Liver cirrhosis
qRT-PCR	Quantitative reverse transcription-polymerase chain reaction
R	Receptor
RIN	RNA integrity number
siRNA	Small interfering RNA
THPO	Thrombopoietin
VEGF	Vascular endothelial growth factor
